# Sex Differences in Lipid Metabolism: Implications for Systemic Lupus Erythematosus and Cardiovascular Disease Risk

**DOI:** 10.3389/fmed.2022.914016

**Published:** 2022-05-31

**Authors:** George A. Robinson, Ines Pineda-Torra, Coziana Ciurtin, Elizabeth C. Jury

**Affiliations:** ^1^Division of Medicine, Centre for Rheumatology Research, University College London, London, United Kingdom; ^2^Division of Medicine, Centre for Adolescent Rheumatology Versus Arthritis, University College London, London, United Kingdom; ^3^Division of Medicine, Centre for Cardiometabolic and Vascular Science, University College London, London, United Kingdom

**Keywords:** sex and gender, lipoproteins, autoimmunity, atherosclerosis, SLE

## Abstract

It is known that healthy women during childbearing years have a lower risk of cardiovascular disease (CVD) and coronary heart disease compared to age matched men. Various traditional risk factors have been shown to confer differential CVD susceptibilities by sex. Atherosclerosis is a major cause of CVD and mortality and sex differences in CVD risk could be due to reduced atherogenic low and very low-density lipoproteins (LDL and VLDL) and increased atheroprotective high density lipoproteins (HDLs) in women. In contrast, patients with systemic lupus erythematosus (SLE), a chronic inflammatory disease that predominately affects women, have an increased atherosclerotic and CVD risk. This increased CVD risk is largely associated with dyslipidaemia, the imbalance of atherogenic and atheroprotective lipoproteins, a conventional CVD risk factor. In many women with SLE, dyslipidaemia is characterised by elevated LDL and reduced HDL, eradicating the sex-specific CVD protection observed in healthy women compared to men. This review will explore this paradox, reporting what is known regarding sex differences in lipid metabolism and CVD risk in the healthy population and transgender individuals undergoing cross-sex hormone therapy, and provide evidence for how these differences may be compromised in an autoimmune inflammatory disease setting. This could lead to better understanding of mechanistic changes in lipid metabolism driving the increased CVD risk by sex and in autoimmunity and highlight potential therapeutic targets to help reduce this risk.

## Introduction

Cardiovascular disease (CVD) is the leading cause of mortality worldwide (1, 2). The most common pathogenic process leading to CVD is atherosclerosis, the build-up of lipids and inflammation in the walls of major arteries (atherosclerotic plaque), leading to the narrowing of the interior lumen of the vessel, plaque rupture, thrombosis, and subsequent myocardial infarction or stroke due to the restricted blood flow to the heart or brain, respectively. Importantly, women of a childbearing age have around half the CVD risk compared to age-matched men, and almost a 10-year delay to first myocardial infarction event (3–5). Whilst sex differences in CVD risk are narrowed in older age groups, the CVD-associated death rate among women never exceeds that of men (6, 7). Traditional risk factors of atherosclerosis that could be modified by sex hormones, such as lipid metabolism, are believed to explain these differential outcomes between men and women (8); however, there is a clear need to investigate these sexually dimorphic mechanisms of CVD to improve outcomes for both men and women.

Alternatively, women represent around 80% of all individuals with autoimmune disease, however, patients with autoimmunity have an increased risk of developing CVD through atherosclerosis (9, 10). With this respect, women between the ages of 35–44 with systemic lupus erythematosus (SLE), a chronic inflammatory disease with a 90% female predominance, have a 50 times increased risk of developing coronary artery disease compared to healthy individuals (11). This shows that the impact of SLE dramatically reduces the female CVD protection seen in healthy individuals. Interplay between traditional risk factors and factors associated with autoimmunity, as well as overlapping factors, such as dyslipidaemia (disrupted lipid metabolism) and inflammation, contribute to accelerated atherosclerosis in SLE patients (12–14).

This review aims to discuss differences in lipid metabolism between men and women, and why this is altered in autoimmunity leading to reduced CVD protection for women. This will aid understanding of the CVD bias by sex and could help to tailor sex specific therapeutic strategies to improve CVD outcomes for both men and women, including those with autoimmunity.

## Sex Differences in Lipoprotein Metabolism: Implications for Cardiovascular Risk

**FIGURE 1:**
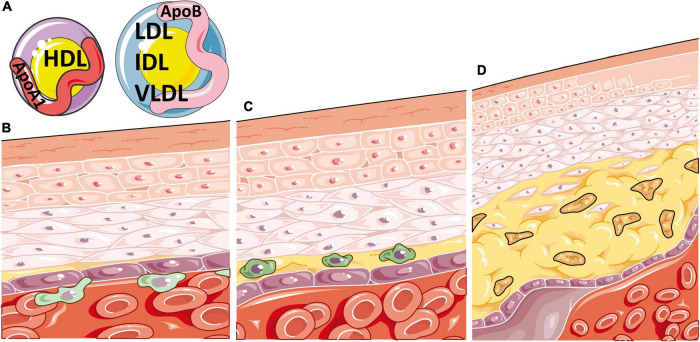
Atherosclerosis is a disease of inflammation and lipids. **(A)** Atherosclerosis is heavily determined by the circulating balance between atheroprotective high density lipoproteins [HDL, expressing apolipoprotein (Apo)A1 on their surface] or atherogenic very low, low and intermediate density lipoproteins (VLDL, LDL and IDL, expressing ApoB on their surface). **(B)** Atherosclerosis initiates when ApoB containing lipoproteins accumulate, become oxidised, and enter the intima region of the blood vessel. This induces endothelial adhesion molecule expression and inflammatory cell recruitment, which migrate through the vessel wall, beginning the process of atherosclerotic plaque formation. **(C)** Oxidised ApoB containing atherogenic lipoproteins are taken up by macrophages in atherosclerotic plaques through scavenger receptors, increasing their cellular lipid burden and resulting in foam cell formation. **(D)** These lipid laden macrophages enlarge the plaque and produce pro-inflammatory cytokines, resulting in further immune recruitment to the plaque, damage to smooth muscle and endothelial cells, necrotic core formation from the growing mass of extracellular lipids and cell debris, narrowing of the artery and eventual thrombosis. This figure was produced using resources from Servier Medical Art, licenced under a Creative Common Attribution 3.0 Generic License. http://smart.servier.com/.

The build-up of lipids in atherosclerotic plaques is largely due to lipoproteins, biochemical assemblies of lipids and apolipoproteins that are structured to enable hydrophobic lipids to transport freely around the blood. Lipoprotein subtypes are defined by their size, density, lipid content and specific apolipoprotein (Apo) expressed on their surface, which together determine their pathogenic contribution to atherosclerosis. Lipoproteins of lower density, including very low, low, and intermediate density lipoproteins (VLDL, LDL, IDL), predominately express ApoB on their surface and promote lipid uptake by inflammatory cells in atherosclerotic plaques following their oxidation. Alternatively, high density lipoproteins (HDLs) express ApoA1 on their surface and play a role in lipid efflux, inferring a role that is typically atheroprotective (15) (Figure 1). Emerging research supports that different sizes of lipoprotein sub-classes can infer differential effects on CVD risk (16), highlighting the need for more detailed analytical methods for serum lipid profiling, such as nuclear magnetic resonance (NMR) spectroscopy, to expand the standard lipid fraction routinely measured in clinical practice (LDL-cholesterol, HDL-cholesterol, total-cholesterol, and total triglycerides, TGs).

It is well established that prior to menopause, the lipoprotein profile of healthy women is more atheroprotective compared to age matched men (17). The Framingham Offspring Study is one of the largest studies to investigate sex differences in CVD risk factors, where subsequent interrogation of this data has identified an increase in smaller and more dense LDL particles in men compared to women (18, 19), a subset that has been previously associated with sex differences in CVD incidence (20–22). Since these observations, NMR spectroscopy analysis of serum from 1574 men and 1692 women (mean age of 52 years) from the Framingham Offspring Study confirmed the lower CVD risk lipid profile in women, where women had a twofold higher concentration of large HDL particles compared to men (23). Large-HDL subsets have been shown to confer higher CVD protection (16). Complimentary to previous studies, the difference in HDL particle size between men and women decreased with age in the Framingham Offspring Study cohort. Furthermore, previously established differences in conventional lipid measures, with men having higher concentrations of TGs, LDL-cholesterol and ApoB, but lower HDL-cholesterol and ApoA1, were also confirmed (23). Importantly, VLDL particles have a high content of TGs relative to other lipoproteins classes, and have also been associated with residual CVD risk independent of circulating TGs (24–26), however, this subset has been less well studied due to the focus of clinical lipid profiles on LDL and HDL-cholesterol measures.

Following menopause, women lose a large amount of their protective lipoprotein fractions which is reflected in increased CVD post-menopause. This is believed to be a result of reduced circulating oestradiol, where lower levels have been shown to infer an increased risk of developing metabolic diseases and CVD (27). A study of post-menopausal women, assessing coronary artery calcification (CAC), a measure of established atherosclerosis using electron beam computed tomography, found that small LDL and all VLDL subclasses were significantly associated with a higher extent of CAC (28). However, large HDL particles, but not small, inversely correlated with the extent of CAC, highlighting the protective role of HDL even in older women with lower oestradiol levels. In support, studies have shown that post-menopause, LDL increases in women to the levels of age-matched men, however, HDL remains higher in women compared to men at all ages despite the decrease post-menopause (29–32).

As heart disease is more common in older age groups and age is an independent risk factor for CVD, studies of CVD are more common in adults (33). However, new studies have explored lipoprotein metabolism in younger age groups, particularly surrounding puberty, where hormones have been shown to become extremely relevant for sexual dimorphisms in CVD risk factors. With this respect, Robinson et al. recently explored sex differences in detailed lipoprotein profiles using NMR metabolomics of serum from young, healthy pre- and post-pubertal individuals (34). This study showed that pre-puberty, no differences in lipoproteins exist, however, following the onset of puberty (assessed clinically using standardised Tanner stages), young men develop an atherogenic profile, consisting mostly of increased larger VLDL subsets and VLDL lipid content, whilst young women develop an increase in total, medium and larger HDL particles, HDL lipid content and levels of ApoA1. In addition, this study performed detailed serum lipoprotein profiling of a rare cohort of young transgender individuals, which validated the direct association between oestradiol and increased larger HDL and ApoA1 levels in trans-women (young individuals born phenotypically male, who were treated with puberty blockers followed by oestradiol, as gender reaffirming therapy), as well as between testosterone and increased VLDL levels in trans-men (young individuals born phenotypically female, who were treated with puberty blockers followed by testosterone, as gender reaffirming therapy). As supported by these studies of different age groups, this suggests that VLDL versus LDL could be the dominantly increased atherogenic lipoproteins in younger versus older adult men compared to age matched women. Importantly, increased circulating concentrations of LDL and VLDL in plasma have been shown to induce the development of atherosclerosis, independent of other risk factors (35). Finally, this study showed that HDL was increased by oestradiol in a dose dependent and chromosome independent manner in trans-women, suggesting that HDL may be more sensitive to changing hormones levels than atherogenic lipoproteins at this young age. Sex-specific changes in lipoproteins discussed are summarised in Table 1.

**TABLE 1:** Sex differences in lipoproteins across pubertal stages.

	Pre-puberty (girls versus boys)	Post-puberty (women versus men)	Post-menopause (women versus age matched men)
ApoB	• No difference (34)	• Increased in men (23)	
VLDL	• No difference (34)	• Increased in men (34)	
LDL	• No difference (34)	• Increased in men (23) • Increased small-LDL in men (18, 19)	• Increased in women (29–31)
ApoA1	• No difference (34)	• Increased in women (23, 34)	
HDL	• No difference (34)	• Increased in women (23, 34) • Twofold higher large-HDL in women (23)	• Lower lipid rich HDL than pre-menopause, but still increased in women (29–32)

**FIGURE 2:**
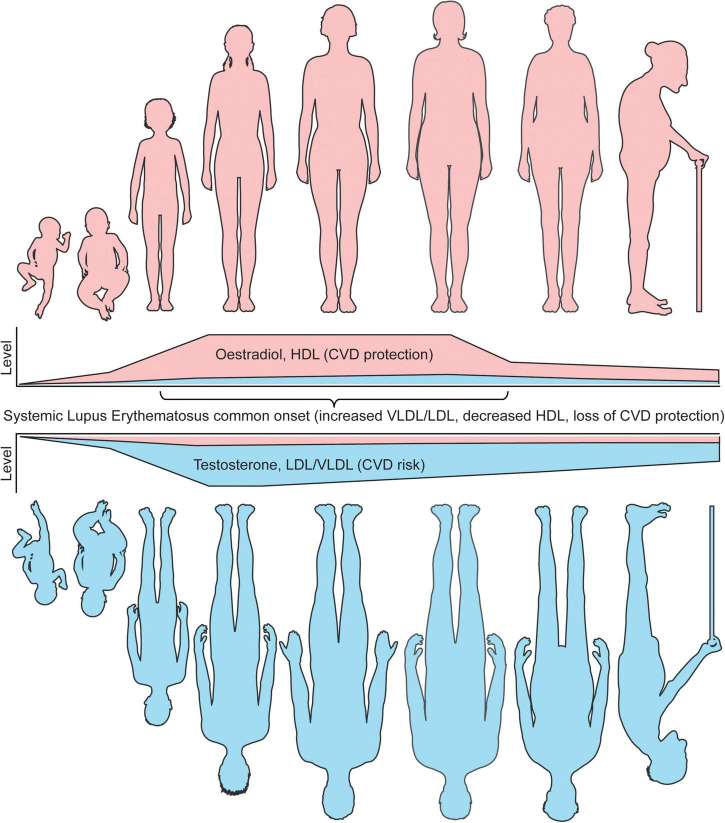
Sex differences in lipoproteins and CVD risk coincide with age associated hormone changes. The levels of circulating sex hormones change throughout life. Pre-puberty, research has shown that girls and boys do not have differences in either atherogenic (VLDL/LDL) or atheroprotective (HDL) lipoproteins. Following the onset of puberty, oestradiol increases in young women, which in turn raises the level of circulating HDL, inferring a lower CVD risk compared to young men in healthy individuals. Post-pubertal young men, with increased testosterone and low oestradiol levels, develop a more atherogenic lipoprotein profile, inferring an increased CVD risk compared to young women in healthy individuals. Whilst age is an independent risk factor of CVD risk in both men and women, oestradiol and HDL remain high in women until menopause, where oestradiol dramatically reduces and CVD protection by HDL is less prominent. Despite this, the levels of HDL in women remain higher than men post-menopause. Older men do not experience this dramatic fall in sex hormones, however, the levels of testosterone do slowly reduce with age. A recent study has shown that sex hormone associated lipoprotein changes can be induced by cross sex hormones in young transgender individuals, supporting these CVD risk associated observations. In patients with SLE, a disease with most common onset in women of a childbearing age, where women represent around 90% of all patients, CVD protection in women is dramatically reduced due to dyslipideamia. This includes increased atherogenic lipoproteins and reduced HDL. It is speculated that this could be due to changes in levels or tissue sensitivity oestradiol, which drives inflammation and altered lipid metabolism. Understanding these fundamental differences in lipoproteins by sex will aid our mechanistic understanding of sexually dimorphic diseases and improve disease prevention and outcomes for CVD and autoimmune patients. This figure was produced using resources from Servier Medical Art, licenced under a Creative Common Attribution 3.0 Generic License. http://smart.servier.com/.

Together, these studies highlight that sex differences in atherosclerosis susceptibilities could be inferred from a young age by hormones and supports a role of hormones in driving lipoprotein metabolism at both ends of the age scale, as well as the importance of studying lipoproteins and CVD susceptibilities at all ages and genders (Figure 2).

## Lipoprotein Metabolism and Dyslipidaemia in Women With Systemic Lupus Erythematosus

SLE is a complex and heterogeneous autoimmune disorder characterised by loss of immune cell regulation, chronic inflammation, and multiple organ damage. As well as genetic, environmental, and epigenetic contributions, hormones have also been implicated in the aetiology of SLE due to the sexual dysmorphism of the disease, where the female to male ratio is 9:1 (36). Deaths attributable to disease activity in SLE have reduced dramatically over the last 50 years due to improved treatments targetting key dysregulated immune pathways, however, deaths associated with atherosclerosis and CVD are still high (37, 38). It has become apparent that the pathogenesis of atherosclerosis shares several autoimmune inflammatory pathways (39). Aside from inflammation, dyslipideamia (an imbalance between atherogenic and atheroprotective lipoproteins) is extremely common in SLE and is a conventional CVD-risk factor through atherosclerosis (40). In fact, dyslipidemia was found in over 70% of premature coronary heart disease cases and hypercholesterolaemia (elevated total and/or LDL/non-HDL-cholesterol) was present in 34–51% of SLE all patients (41). In addition, a Systemic Lupus International Collaborating Clinics’ (SLICC) cohort study reported that 36% of newly diagnosed SLE patients had hypercholesterinemia in this large international cohort, which increased to over 60% after 3 years (42, 43). Interplay between traditional CVD risk factors, including dyslipidaemia, and risk factors associated with ongoing chronic inflammation captured in disease activity scores and cumulative steroid treatment (the most used treatment for acute flares in SLE) could contribute to the accelerated development of atherosclerosis in men and women with SLE (12). Whilst healthy women of a childbearing age typically have a more atheroprotective lipoprotein profile compared to men, as the onset of SLE peaks between the ages of 15–55 and the disease has a significant female-bias (44), the atheroprotective lipid profile is replaced by dyslipideamia which is common in all patients with lupus (Figure 2 depicts the physiological variation of lipid profile in men versus women; their associations with sex hormones and age, as well as impact of SLE-related chronic inflammation and treatment on driving a dysregulated lipid profile in all patients). Various clinical studies have found that elevated total cholesterol, TGs, circulating LDL-cholesterol and reduced HDL-cholesterol are the most common lipid abnormalities associated with SLE (40), which likely contributes to the higher lipid burden of atherosclerotic plaques, and increased CVD-risk in many patients. However, these standard clinical lipid panels, as well as many established cardio-vascular risk scores, often fail to account for the increased CVD-risk directly associated with SLE disease and treatment, and studies often do not consider how the presence of SLE modifies the impact of sex on CVD-risk (14, 45, 46). Despite this, more detailed investigations into lipoprotein subsets using NMR technology have found that women with SLE have increases in smaller LDL subfractions compared to sex matched healthy controls (HCs) (47). Further to this, a serum NMR metabolomic study by Coelewij et al., incorporating detailed lipoprotein subclass evaluation, was able to confidently differentiate between adult women with SLE and sex matched HCs by use of machine learning (48). Here, the most influential metabolites in separating SLE from HCs were medium sized HDL measures, which were reduced in SLE, as well as small HDL, VLDL, and IDL particles, which were increased in SLE compared to HCs. This suggests that different HDL sizes are important to consider when studying dyslipidaemia in SLE. In support, another study reported that smaller HDL subsets were reduced in SLE, whilst no difference in the size of VLDL or LDL were reported (49). Dyslipidaemia has also been identified by multiple studies of paediatric patients with juvenile-SLE (50, 51), where onset of SLE occurs before the age of 18 and patients typically have worse disease outcomes and an estimated 100- to 300-fold increased risk of mortality from CVD compared to age-matched healthy individuals (52, 53). Strikingly, dyslipidaemia is present in up to 63% of patients with juvenile-SLE, which is higher in patients with active disease (51). In addition, an NMR metabolomics study of these younger juvenile-SLE patients showed that small HDL subsets were the most significantly reduced lipoproteins in juvenile-SLE patients compared to HCs, validated by machine learning analysis; this reduction was also exacerbated by increased disease activity (54). Importantly, sex was adjusted for in this analysis, despite 81.5% of this cohort being female. In a more sex-specific study of young girls with juvenile-SLE, dyslipidaemia was observed in 39% of the study participants and a significant decrease in HDL-associated ApoA1 the juvenile-SLE cohort compared to HCs, supporting a more global decrease in HDL in young patients (55).

Based on available literature data, there is compelling evidence that dyslipidaemia associated with SLE could dramatically reduce the lipid protection that healthy women of a childbearing age have from CVD, even in much younger age groups. This was supported recently by Robinson et al., who investigated sex differences in lipoprotein metabolism between young, post-pubertal patients with juvenile-SLE and found that all conventional differences in lipoprotein profiles observed between age-matched healthy men and women were lost in patents with juvenile-SLE (34). A sex-specific sub-analysis showed an increase in VLDL subsets and a decrease in HDL subsets in young women with juvenile-SLE compared to HCs, supporting reduced atheroprotection in disease. This loss of protection could be due to a breakdown in conventional sex hormone signalling, and highlights that sex and age are extremely important when studying the pathogenesis of and associated dyslipidaemia in SLE, where additional factors, such as ongoing inflammation and differential sex hormones are likely to have a significant impact on the overall CVD risk. Sex differences in lipid metabolism and their impact on the CVD-risk of patients with SLE are not commonly studied due to the overwhelming female predominance of the disease; however, this needs to be a priority going forward to enable better understand of the changes in CVD risk for women with SLE of all ages.

## Discussion

**FIGURE 3:**
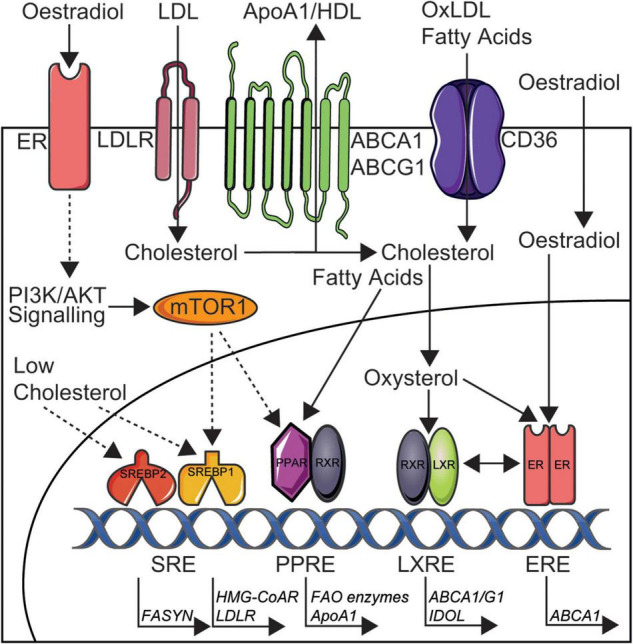
Simplified depiction of oestradiol induced signalling pathways that may increase cardioprotection in women through altered lipid metabolism and could be targeted in SLE. Cholesterol is taken up by cells in low density lipoproteins (LDL) *via* LDL receptors (LDLR) and CD36 [oxidised (ox)LDL] or is synthesised by HMG-CoA reductase (HMG-CoAR). Cholesterol is removed from cells to ApoA1/high density lipoproteins (HDL) *via* ATP-binding cassette transporter A1 or G1 (ABCA1/G1) or converted to oxysterols intracellularly. Low intracellular cholesterol levels can induce the transcriptional activity of sterol regulatory element-binding proteins 1/2 (SREBP1/2). Fatty acids are taken up by CD36, synthesised by fatty acid synthase (FASYN), or metabolised by fatty acid oxidation (FAO). Oestradiol acts *via* membrane bound oestrogen receptors (ERs) to induce phosphoinositide 3-kinase/protein kinase B (PI3K/AKT) signalling and downstream mTOR1 activation. Activated mTOR1 increases lipogenesis *via* increased nuclear activity of SREBP1 and peroxisome proliferator-activated receptor (PPAR) transcription factors. Oestradiol can also activate transcription factor ERs in the nucleus and this increased ER nuclear activity can elevate the binding and transcriptional efficiency of liver-x-receptors (LXRs), inducing cardioprotective cholesterol metabolism through upregulated ABCA1/G1 and inducible degrader of the LDLR (IDOL). It is through these metabolic pathways that oestradiol could exert it’s cardioprotective effects in healthy women and therefore could be disrupted and targetted in SLE to reduce CVD risk for patients. This figure was produced using resources from Servier Medical Art, licenced under a Creative Common Attribution 3.0 Generic License. http://smart.servier.com/.

Is it striking that the presence of SLE in women removes the sex-specific cardio-protection through dyslipidaemia and this highlights a possible role for deregulated oestradiol signalling in SLE, in addition to over-activation of proinflammatory pathways and impact of certain SLE medications on lipid metabolism, all ultimately leading to altered lipid profiles in these patients. The association between lipids and sex-hormones is not a new theory, where the combined oral contraceptive pill (oestradiol and progesterone) has been previously shown to increase circulating HDL-cholesterol and TGs, whilst the progesterone only pill has no effect (56). Oestradiol administration has also been shown to increase HDL in post-menopausal women (57, 58), supporting a direct cardioprotective role of sex hormones in lipid metabolism. The study by Robinson et al. outlined above (34), highlighted that trans-men had increased total and LDL-cholesterol and TGs as well as decreased HDL-cholesterol associated to short-term administration of exogenous testosterone as gender-reaffirming treatment (and reduced oestradiol following treatment with puberty blockers), whilst trans-women had decreased total and LDL-cholesterol associated with exposure to short-term therapeutic oestradiol doses (in the context of reduced testosterone following treatment with puberty blockers) (59, 60). Follow up studies will be critical to understand the long-term effects of these sex hormone and lipid changes on CVD risk, as well as larger cohort studies to control for confounding factors such as BMI, hypertension and smoking. Studies in mice have also supported a protective role of oestradiol in CVD through lipid metabolism, where different stages of the menstrual cycle determine the size and lipid content of HDL produced by hepatic cells *in vivo*, relative to the levels of circulating oestradiol (61). When oestradiol levels are highest, smaller HDL particles are produced which allow more efficient cholesterol efflux from the liver *via* ATP-binding cassette transporter A1/G1 (ABCA1/G1), which in turn infers greater CVD protection. This is due to the increased nuclear activity of the oestrogen receptor, which elevates the binding and transcriptional efficiency of liver-x-receptors (ABCA1/G1), master regulators of cellular cholesterol metabolism (Figure 3). Where smaller HDL particles are more efficient in mice regarding cholesterol efflux, it has been alternatively shown that large- and medium-sized HDL-cholesterol is more protective of myocardial infarction and stroke in humans (16), suggesting complex differences across models. *De novo* clearance of LDL and VLDL is also increased when plasma oestradiol levels are high in mouse models (61, 62), suggesting a duel effect of oestradiol on increasing and decreasing atheroprotective and atherogenic lipoproteins, respectively. Conversely, testosterone has been shown to increase hepatic lipase activity (catalyses the hydrolysis of triglycerides), decrease the levels of HDL and reduce the size of LDL (63). Reports on the specific impact of testosterone on atherosclerosis and CVD is less researched compared to studies of oestradiol (64).

Together, these sex specific lipid changes may explain why women lose their CVD protection following menopause, however, may not explain why women of a childbearing age with SLE develop an increased CVD risk. Although a reduction in oestradiol induced signalling could be a logical explanation for the increase CVD risk in SLE, in fact, many studies have reported that oestradiol and the oestrogenic metabolite, 6 α-hydroxyestrone, are increased in women with SLE (65–68), supporting the predominance of SLE disease onset in women during their reproductive years. Alternatively, it is plausible that oestradiol may promote inflammation in SLE, which increases the impact of non-traditional CVD risk factors including chronic inflammation (36). In support, some reports show that inflammatory flares in SLE are more prominent during pregnancy (69) and that patients with SLE may have exaggerated inflammatory responses to oestradiol (70). Generalised inflammation can reduce HDL levels and increase hepatic VLDL production, whilst reducing the clearance of TG lipoproteins (71). More specifically, inflammation in SLE associated with disease flares and pro-inflammatory cytokines such as IL-6 and TNF-α can increase TG and reduce HDL levels (72, 73). This could be partly due to reduced cell-mediated cholesterol efflux in SLE (74). Larger, TG rich lipoproteins have been associated with a duel effect on inflammation and atherosclerosis, whereas smaller LDL particles can promote atherosclerosis independent of inflammation (75). In addition, VLDL particles may also have difficulty leaving the subendothelial space of blood vessels, promoting local inflammation and atherosclerotic plaque progression (76). Finally, altered liver function, the major regulator of systemic lipid metabolism, is more common in SLE due to inflammation (77–79). This liver inflammation, along with current therapies used to treat SLE, such as steroids, could contribute to the loss of atheroprotection in women through altered lipoprotein metabolism, while treatment with hydroxychloroquine which is currently recommended in all patients with SLE can counterbalance some of the negative effects SLE has on the CVD-risk profile of these patients (80).

Another master regulator of metabolism that also has an impact on lipid synthesis is the mammalian target of rapamycin (mTOR) (81). Specifically, upon stimulation through phosphoinositide 3-kinase/protein kinase B (PI3K/AKT) signalling, mTORC1 inhibits lipolysis and induces lipogenesis *via* the activation of peroxisome proliferator-activated receptor γ (PPARγ) and sterol regulatory element-binding transcription factor 1 (SREBP1) (82, 83). Separately, activated mTORC2 can induce AKT signalling and therefore mTORC1 activation indirectly. With respect to these mechanisms, liver-specific deletion of mTORC1 can induce cardioprotective effects and render mice resistant to western diet induced hypercholesterolaemia (82, 84). mTOR inhibition with rapamycin has also led to a significant reduction of atherosclerotic lesions in LDL-receptor (LDLR) deficient male mice, despite severe hypercholesterolaemia (85). Despite these beneficial effects of mTOR inhibition on cardiovascular health, mTORC1 inhibition can also cause dyslipidaemia, a common risk factor for atherosclerosis, through downregulation of hepatic LDLRs and stimulation of lipophagy, resulting in a respective increase in circulating levels of LDL-cholesterol and droplet-released lipids (86, 87), meaning that the overall contribution of mTOR to atherosclerotic risk is complex (88). To add to this complexity, sex differences in mTOR signalling have been described in mouse models, where oestradiol stimulation of the oestrogen receptor can induce PI3K/AKT signalling and downstream mTOR activation (89, 90). mTOR suppression with rapamycin can increase the lifespan of mice (91), leading to an interest in its sex-specific effects on cardiovascular health. With this respect, increased mTORC1 activity has been observed in the liver and heart tissue of young female mice compared to male mice of the same age (92), and rapamycin treatment in mice also has sex specific effects on mTORC1 and mTORC2 (93). mTOR inhibition has also been shown to improve testosterone-induced myocardial hypertrophy in hypertensive rats (94), together supporting the potential sex-specific effects of mTOR on cardiovascular health. This suggests an alternative metabolic pathway to liver-x-receptors that oestradiol may exert its cardioprotective effects in healthy women and could be disrupted in SLE (Figure 3). It is also reported that patients with SLE have genetic activation of mTORC1 (95), and its blockade exerts potential therapeutic efficacy in SLE through reducing pro-inflammatory T-cell and macrophage differentiation (96). mTOR activation has also been implicated in increased CVD in SLE (97). Various mTOR inhibitors, less or more selective, have been developed for use in cancers and transplant medicine, owing to their important antiproliferative and cellular effects and immunosuppressive effects, although further research is required to address the limitations of dose-related toxicity and lack of tissue selectivity (98). Therefore, the cardiovascular and inflammatory effects of mTOR appear to be model, tissue, disease, and sex specific, adding to the complexity of investigating sex differences in CVD and autoimmune susceptibilities; more human studies are warranted.

## Conclusion and Perspectives

Whilst CVD is more common in men, and SLE in women, sex and gender needs to be taken into account in all medical research. With this respect, CVD is the leading cause for mortality in women, representing 35% of all global deaths (99). According to a recent study by The Lancet, 275 million women were diagnosed with CVD and 8.9 million died from CVD in 2019 (100). Despite this, women are hugely under-represented in clinical trials of CVD due to the increased risk in men, which is a major global health research limitation which needs to be addressed. Not only this, but women are often under-researched, underdiagnosed and undertreated as a result of this sex bias in CVD risk. In response, The Lancet have produced The Lancet women and CVD commission, aiming to reducing the global burden of CVD on women by 2030 (101). Here, an all-female led commission outlined new recommendations to tackle inequities in diagnosis, treatment, and prevention to reduce CVD in women. With regard to lipids, a standardised case-control study of acute myocardial infarction across 52 countries, the INTERHEART study, showed that abnormal lipids were the highest population attributable risk factor for CVD with very little contributable difference between sexes (49.5% for men and 47.1% for women) (8), validating the importance of considering both sexes in CVD, particularly when studying lipid metabolism.

It is also important to note that men are underrepresented in studies and clinical trials of SLE (102). Relative to CVD, however, SLE is relatively rare in the general population and men only represent around 10% of all SLE cases. This makes equality in SLE research difficult, particularly regarding sex, where men only represent around 7% of randomised controlled trials of patients with SLE (102). Despite this, it has been shown that men with SLE tend to develop more severe renal manifestations and higher risk of end-stage renal disease, requiring increased monitoring in clinical practice (103). With this respect, men should be considered more in research and clinical trials of SLE to improve disease prognosis. As highlighted in this review, equality in research cohorts and clinical trials not only improves lives of men and women, but also helps us to understand the pathogenic mechanisms of sexually dimorphic diseases.

To conclude, whilst oestradiol conventionally promotes atheroprotective lipoprotein metabolism in healthy individuals, chronic inflammation due to altered oestradiol sensitivity in patients with SLE, as well as other SLE-related treatment factors could alter finely tuned mechanisms of lipid regulation and induce circulating lipoprotein changes toward a more atherogenic profile. It is clear that further mechanistic investigations are warranted, however, uncovering these mechanisms of fundamental sex hormone driven changes in lipid metabolism will aid disease prevention and outcomes for both patients with CVD and autoimmunity, regardless of sex or gender, highlighting the importance of considering sex hormones in medical research.

## Author Contributions

GR performed the literature review and wrote the first draft of the manuscript. All authors reviewed the manuscript and approved the final version.

## Author Disclaimer

The views expressed are those of the authors and not necessarily those of the NHS, the NIHR, or the Department of Health.

## Conflict of Interest

The authors declare that the research was conducted in the absence of any commercial or financial relationships that could be construed as a potential conflict of interest.

## Publisher’s Note

All claims expressed in this article are solely those of the authors and do not necessarily represent those of their affiliated organizations, or those of the publisher, the editors and the reviewers. Any product that may be evaluated in this article, or claim that may be made by its manufacturer, is not guaranteed or endorsed by the publisher.

## References

[1] Lozano R Naghavi M Foreman K AlMazroa MA Memish ZA. Global and regional mortality from 235 causes of death for 20 age groups in 1990 and 2010: a systematic analysis for the global burden of disease study 2010 (vol 380, pg 2095, 2012). *Lancet.* (2013) 381:628. 10.1016/S0140-6736(12)61728-0 23245604 PMC10790329

[2] Mc Namara K Alzubaidi H Jackson JK. Cardiovascular disease as a leading cause of death: how are pharmacists getting involved? *Integr Pharm Res Pract.* (2019) 8:1–11. 10.2147/IPRP.S133088 30788283 PMC6366352

[3] Roger VL Go AS Lloyd-Jones DM Adams RJ Berry JD Brown TM Executive summary: heart disease and stroke statistics-2011 update a report from the American heart association. *Circulation.* (2011) 123:459–63. 10.1161/CIR.0b013e3182009701 PMC4418670 21160056

[4] Lloyd J. Heart disease and stroke statistics-2009 update: a report from the American heart association statistics committee and stroke statistics subcommittee (vol 119, pg e21, 2009). *Circulation.* (2011) 124:E424–E. 10.1161/CIRCULATIONAHA.108.191261 19075105

[5] Wilmot KA O’Flaherty M Capewell S Ford ES Vaccarino V. Coronary heart disease mortality declines in the United States from 1979 through 2011 evidence for stagnation in young adults especially women. *Circulation.* (2015) 132:997–1002. 26302759 10.1161/CIRCULATIONAHA.115.015293 PMC4828724

[6] Kannel WB Wilson PWF. Risk-factors that attenuate the female coronary-disease advantage. *Arch Intern Med.* (1995) 155:57–61. 7802521

[7] Price JF Fowkes GR. Risk factors and the sex differential in coronary artery disease. *Epidemiology.* (1997) 8:584–91. 10.1097/00001648-199709000-00018 9270963

[8] Yusuf S Hawken S Ounpuu S. Effect of potentially modifiable risk factors associated with myocardial infarction in 52 countries (the INTERHEART study): case-control study (vol 364, pg 937m 2004). *Lancet.* (2004) 364:2020. 10.1016/S0140-6736(04)17018-9 15364185

[9] Sherer Y Shoenfeld Y. Mechanisms of disease: atherosclerosis in autoimmune diseases. *Nat Clin Pract Rheumatol.* (2006) 2:99–106. 10.1038/ncprheum0092 16932663

[10] McCarthy M. The “gender gap” in autoimmune disease. *Lancet.* (2000) 356:1088. 10.1016/S0140-6736(05)74535-9 11009154

[11] Manzi S Meilahn EN Rairie JE Conte CG Medsger TA Jr Jansen-McWilliams L Age-specific incidence rates of myocardial infarction and angina in women with systemic lupus erythematosus: comparison with the Framingham study. *Am J Epidemiol.* (1997) 145:408–15. 10.1093/oxfordjournals.aje.a009122 9048514

[12] Schanberg LE Sandborg C Barnhart HX Ardoin SP Yow E Evans GW Premature atherosclerosis in pediatric systemic lupus erythematosus: risk factors for increased carotid intima-media thickness in the atherosclerosis prevention in pediatric lupus erythematosus cohort. *Arthritis Rheum.* (2009) 60:1496–507. 10.1002/art.24469 19404953 PMC2770725

[13] Barsalou J Bradley TJ Silverman ED. Cardiovascular risk in pediatric-onset rheumatological diseases. *Arthritis Res Ther.* (2013) 15:12. 10.1186/ar4212 23731870 PMC3672705

[14] Bruce IN. ‘Not only.but also’: factors that contribute to accelerated atherosclerosis and premature coronary heart disease in systemic lupus erythematosus. *Rheumatology.* (2005) 44:1492–502. 10.1093/rheumatology/kei142 16234277

[15] Arsenault BJ Boekholdt SM Kastelein JJP. Lipid parameters for measuring risk of cardiovascular disease. *Nat Rev Cardiol.* (2011) 8:197–206. 10.1038/nrcardio.2010.223 21283149

[16] Holmes MV Millwood IY Kartsonaki C Hill MR Bennett DA Boxall R Lipids, lipoproteins, and metabolites and risk of myocardial infarction and stroke. *J Am Coll Cardiol.* (2018) 71:620–32. 10.1016/j.jacc.2017.12.006 29420958 PMC5811927

[17] Hazzard WR. Atherogenesis – why women live longer than men. *Geriatrics.* (1985) 40:42–51, 54. 3965355

[18] McNamara JR Campos H Ordovas JM Peterson J Wilson PWF Schaefer EJ. Effect of gender, age, and lipid status on low-density-lipoprotein subfraction distribution – results from the Framingham offspring study. *Arteriosclerosis.* (1987) 7:483–90. 10.1161/01.atv.7.5.483 3675308

[19] Campos H Blijlevens E McNamara JR Ordovas JM Posner BM Wilson PWF LDL particle-size distribution – results from the Framingham offspring study. *Arterioscler Thromb.* (1992) 12:1410–9. 10.1161/01.atv.12.12.1410 1450174

[20] Gardner CD Fortmann SP Krauss RM. Association of small low-density lipoprotein particles with the incidence of coronary artery disease in men and women. *JAMA.* (1996) 276:875–81. 8782636

[21] Chung CP Oeser A Raggi P Solus JF Avalos I Linton MF Lipoprotein subclasses and particle size determined by nuclear magnetic resonance spectroscopy in systemic lupus erythematosus. *Clin Rheumatol.* (2008) 27:1227–33. 10.1007/s10067-008-0890-4 18421545

[22] Kuller L Arnold A Tracy R Otvos J Burke G Psaty B Nuclear magnetic resonance spectroscopy of lipoproteins and risk of coronary heart disease in the cardiovascular health study. *Arterioscler Thromb Vasc Biol.* (2002) 22:1175–80. 10.1161/01.atv.0000022015.97341.3a 12117734

[23] Freedman DS Otvos JD Jeyarajah EJ Shalaurova I Cupples LA Parise H Sex and age differences in lipoprotein subclasses measured by nuclear magnetic resonance spectroscopy: the Framingham study. *Clin Chem.* (2004) 50:1189–200. 10.1373/clinchem.2004.032763 15107310

[24] Freedman DS Otvos JD Jeyarajah EJ Barboriak JJ Anderson AJ Walker JA. Relation of lipoprotein subclasses as measured by proton nuclear magnetic resonance spectroscopy to coronary artery disease. *Arterioscler Thromb Vasc Biol.* (1998) 18:1046–53. 10.1161/01.atv.18.7.1046 9672064

[25] Prenner SB Mulvey CK Ferguson JF Rickels MR Bhatt AB Reilly MP. Very low density lipoprotein cholesterol associates with coronary artery calcification in type 2 diabetes beyond circulating levels of triglycerides. *Atherosclerosis.* (2014) 236:244–50. 10.1016/j.atherosclerosis.2014.07.008 25105581 PMC4209900

[26] Duran EK Pradhan AD. Triglyceride-rich lipoprotein remnants and cardiovascular disease. *Clin Chem.* (2021) 67:183–96. 10.1093/clinchem/hvaa296 33409533

[27] Della Torre S Benedusi V Fontana R Maggi A. Energy metabolism and fertility-a balance preserved for female health. *Nat Rev Endocrinol.* (2014) 10:13–23. 10.1038/nrendo.2013.203 24146033

[28] Mackey RH Kuller LH Sutton-Tyrrell K Evans RW Holubkov R Matthews KA. Lipoprotein subclasses and coronary artery calcium in postmenopausal women from the healthy women study. *Am J Cardiol.* (2002) 90:71I–6I. 10.1016/s0002-9149(02)02636-x 12419483

[29] Matthews KA Meilahn E Kuller LH Kelsey SF Caggiula AW Wing RR. Menopause and risk-factors for coronary heart-disease. *N Engl J Med.* (1989) 321:641–6. 2488072 10.1056/NEJM198909073211004

[30] Abbey M Owen A Suzakawa M Roach P Nestel PJ. Effects of menopause and hormone replacement therapy on plasma lipids, lipoproteins and LDL-receptor activity. *Maturitas.* (1999) 33:259–69. 10.1016/s0378-5122(99)00054-7 10656504

[31] Kannel WB. Citation classic – serum-cholesterol, lipoproteins, and the risk of coronary heart-disease – the Framingham-study. *Curr Contents Life Sci.* (1983) 29:18. 10.7326/0003-4819-74-1-1 5539274

[32] Stevenson JC Crook D Godsland IF. Influence of age and menopause on serum-lipids and lipoproteins in healthy women. *Atherosclerosis.* (1993) 98:83–90. 10.1016/0021-9150(93)90225-j 8457253

[33] Rodgers JL Jones J Bolleddu SI Vanthenapalli S Rodgers LE Shah K Cardiovascular risks associated with gender and aging. *J Cardiovasc Dev Dis.* (2019) 6:19. 10.3390/jcdd6020019 31035613 PMC6616540

[34] Robinson GA Peng J Peckham H Radziszewska A Butler G Pineda-Torra I Sex hormones drive changes in lipoprotein metabolism. *iScience.* (2021) 24:103257. 10.1016/j.isci.2021.103257 34761181 PMC8567005

[35] Skalen K Gustafsson M Rydberg EK Hulten LM Wiklund O Innerarity TL Subendothelial retention of atherogenic lipoproteins in early atherosclerosis. *Nature.* (2002) 417:750–4. 10.1038/nature00804 12066187

[36] Klein SL Flanagan KL. Sex differences in immune responses. *Nat Rev Immunol.* (2016) 16:626–38. 27546235 10.1038/nri.2016.90

[37] Bernatsky S Boivin J-F Joseph L Manzi S Ginzler E Gladman DD Mortality in systemic lupus erythematosus. *Arthritis Rheum.* (2006) 54:2550–7. 16868977 10.1002/art.21955

[38] Nossent J Cikes N Kiss E Marchesoni A Nassonova V Mosca M Current causes of death in systemic lupus erythematosus in Europe, 2000—2004: relation to disease activity and damage accrual. *Lupus.* (2007) 16:309–17. 10.1177/0961203307077987 17576731

[39] Jara LJ Medina G Vera-Lastra O Amigo MC. Accelerated atherosclerosis, immune response and autoimmune rheumatic diseases. *Autoimmun Rev.* (2006) 5:195–201. 10.1016/j.autrev.2005.06.005 16483919

[40] Szabo MZ Szodoray P Kiss E. Dyslipidemia in systemic lupus erythematosus. *Immunol Res.* (2017) 65:543–50. 28168401 10.1007/s12026-016-8892-9

[41] Wajed J Ahmad Y Durrington PN Bruce IN. Prevention of cardiovascular disease in systemic lupus erythematosus - proposed guidelines for risk factor management. *Rheumatology.* (2004) 43:7–12. 10.1093/rheumatology/keg436 12867578

[42] Urowitz MB Gladman D Ibanez D Fortin P Sanchez-Guerrero J Bae S Clinical manifestations and coronary artery disease risk factors at diagnosis of systemic lupus erythematosus: data from an international inception cohort. *Lupus.* (2007) 16:731–5. 10.1177/0961203307081113 17728367

[43] Urowitz MB Gladman D Ibanez D Fortin P Sanchez-Guerrero J Bae S Accumulation of coronary artery disease risk factors actors over three years: data from an international inception cohort. *Arthritis Rheum Arthritis Care Res.* (2008) 59:176–80. 10.1002/art.23353 18240193

[44] Angum F Khan T Kaler J Siddiqui L Hussain A. The prevalence of autoimmune disorders in women: a narrative review. *Cureus.* (2020) 12:e8094. 10.7759/cureus.8094 32542149 PMC7292717

[45] Drosos GC Konstantonis G Sfikakis PP Tektonidou MG. Underperformance of clinical risk scores in identifying vascular ultrasound-based high cardiovascular risk in systemic lupus erythematosus. *Eur J Prev Cardiol.* (2021) 28:346–52. 33891687 10.1093/eurjpc/zwaa256

[46] Ciurtin C Robinson GA Pineda-Torra I Jury EC. Challenges in implementing cardiovascular risk scores for assessment of young people with childhood-onset autoimmune rheumatic conditions. *Front Med.* (2022) 9:814905. 10.3389/fmed.2022.814905 35237628 PMC8883038

[47] Nuttall SL Heaton S Piper MK Martin U Gordon C. Cardiovascular risk in systemic lupus erythematosus – evidence of increased oxidative stress and dyslipidaemia. *Rheumatology.* (2003) 42:758–62. 10.1093/rheumatology/keg212 12730535

[48] Coelewij L Waddington KE Robinson GA Chocano E McDonnell T Farinha F Using serum metabolomics analysis to predict sub-clinical atherosclerosis in patients with SLE. *medRxiv* [Preprint]. (2020). 10.1101/2020.08.11.20172536

[49] Hua X Su J Svenungsson E Hurt-Camejo E Jensen-Urstad K Angelin B Dyslipidaemia and lipoprotein pattern in systemic lupus erythematosus (SLE) and SLE-related cardiovascular disease. *Scand J Rheumatol.* (2009) 38:184–9. 10.1080/03009740802541470 19165647

[50] Ilowite NT Samuel P Ginzler E Jacobson MS. Dyslipoproteinemia in pediatric systemic lupus-erythematosus. *Arthritis Rheum.* (1988) 31:859–63. 3134897 10.1002/art.1780310706

[51] Ardalan K Lloyd-Jones DM Schanberg LE. Cardiovascular health in pediatric rheumatologic diseases. *Rheum Dis Clin North Am.* (2022) 48:157–81. 10.1016/j.rdc.2021.09.006 34798945

[52] Hersh AO Trupin L Yazdany J Panopalis P Julian L Katz P Childhood-onset disease as a predictor of mortality in an adult cohort of patients with systemic lupus erythematosus. *Arthritis Care Res.* (2010) 62:1152–9. 10.1002/acr.20179 20235215 PMC3755501

[53] Ciurtin C Robinson GA Pineda-Torra I Jury EC. Comorbidity in young patients with juvenile systemic lupus erythematosus: how can we improve management? *Clin Rheumatol.* (2022) 41:961–4. 10.1007/s10067-022-06093-3 35178646

[54] Robinson GA Peng JJ Pineda-Torra I Ciurtin C Jury EC. Metabolomics defines complex patterns of dyslipidaemia in juvenile-SLE patients associated with inflammation and potential cardiovascular disease risk. *Metabolites.* (2022) 12:3. 10.3390/metabo12010003 35050125 PMC8779263

[55] Machado D Sarni ROS Abad TTO Silva SGL Khazaal EJB Hix S Lipid profile among girls with systemic lupus erythematosus. *Rheumatol Int.* (2017) 37:43–8. 10.1007/s00296-015-3393-z 26573664

[56] Wang Q Wurtz P Auro K Morin-Papunen L Kangas AJ Soininen P Effects of hormonal contraception on systemic metabolism: cross-sectional and longitudinal evidence. *Int J Epidemiol.* (2016) 45:1445–57. 10.1093/ije/dyw147 27538888 PMC5100613

[57] Walsh BW Spiegelman D Morrissey M Sacks FM. Relationship between serum estradiol levels and the increases in high-density lipoprotein levels in postmenopausal women treated with oral estradiol. *J Clin Endocrinol Metab.* (1999) 84:985–9. 10.1210/jcem.84.3.5571 10084583

[58] Shufelt CL Manson JE. Menopausal hormone therapy and cardiovascular disease: the role of formulation, dose, and route of delivery. *J Clin Endocrinol Metab.* (2021) 106:1245–54. 10.1210/clinem/dgab042 33506261 PMC8063246

[59] Fisher AD. Cross-sex hormone therapy in trans persons is safe and effective at short-time follow-up: results from the European network for the investigation of gender incongruence (vol 11, pg 1999, 2014). *J Sex Med.* (2016) 13:732. 10.1111/jsm.12571 24828032

[60] Jarin J Pine-Twaddell E Trotman G Stevens J Conard LA Tefera E Cross-sex hormones and metabolic parameters in adolescents with gender dysphoria. *Pediatrics.* (2017) 139:e20163173. 10.1542/peds.2016-3173 28557738

[61] Della Torre S Mitro N Fontana R Gomaraschi M Favari E Recordati C An essential role for liver ERα in coupling hepatic metabolism to the reproductive cycle. *Cell Rep.* (2016) 15:360–71. 10.1016/j.celrep.2016.03.019 27050513 PMC4835581

[62] Villa A Torre S Stell A Cook J Brown M Maggi A. Tetradian oscillation of estrogen receptor alpha is necessary to prevent liver lipid deposition. *Proc Natl Acad Sci U.S.A.* (2012) 109:11806–11. 10.1073/pnas.1205797109 22761311 PMC3406808

[63] Herbst KL Amory JK Brunzell JD Chansky HA Bremner WJ. Testosterone administration to men increases hepatic lipase activity and decreases HDL and LDL size in 3 wk. *Am J Physiol Endocrinol Metab.* (2003) 284:E1112–8. 10.1152/ajpendo.00524.2002 12736156

[64] Gencer B Bonomi M Adorni MP Sirtori CR Mach F Ruscica M. Cardiovascular risk and testosterone - from subclinical atherosclerosis to lipoprotein function to heart failure. *Rev Endocrine Metab Disord.* (2021) 22:257–74. 10.1007/s11154-021-09628-2 33616800 PMC8087565

[65] Lahita RG Bradlow HL Kunkel HG Fishman J. Alterations of estrogen metabolism in systemic lupus-erythematosus. *Arthritis Rheum.* (1979) 22:1195–8. 10.1002/art.1780221106 508372

[66] Lahita RG Bradlow HL Kunkel HG Fishman J. Increased 16 alpha-hydroxylation of estradiol in systemic lupus-erythematosus. *J Clin Endocrinol Metab.* (1981) 53:174–8. 10.1210/jcem-53-1-174 7240374

[67] Lahita RG Bradlow HL Fishman J Kunkel HG. Abnormal estrogen and androgen metabolism in the human with systemic lupus-erythematosus. *Am J Kidney Dis.* (1982) 2:206–11. 7102669

[68] Singh RP Bischoff DS. Sex hormones and gender influence the expression of markers of regulatory T cells in SLE patients. *Front Immunol.* (2021) 12:619268. 10.3389/fimmu.2021.619268 33746959 PMC7966510

[69] Meyer O. Making pregnancy safer for patients with lupus. *Joint Bone Spine.* (2004) 71:178–82. 10.1016/S1297-319X(03)00155-6 15182787

[70] Weidler C Harle P Schedel J Schmidt M Scholmerich J Straub RH. Patients with rheumatoid arthritis and systemic lupus erythematosus have increased renal excretion of mitogenic estrogens in relation to endogenous antiestrogens. *J Rheumatol.* (2004) 31:489–94. 14994392

[71] Khovidhunkit W Kim MS Memon RA Shigenaga JK Moser AH Feingold KR Effects of infection and inflammation on lipid and lipoprotein metabolism: mechanisms and consequences to the host. *J Lipid Res.* (2004) 45:1169–96. 10.1194/jlr.R300019-JLR200 15102878

[72] Chung CP Oeser A Solus J Avalos I Gebretsadik T Shintani A Inflammatory mechanisms affecting the lipid profile in patients with systemic lupus erythematosus. *J Rheumatol.* (2007) 34:1849–54. 17659756

[73] Svenungsson E Gunnarsson I Fei GZ Lundberg IE Klareskog L Frostegard J. Elevated triglycerides and low levels of high-density lipoprotein as markers of disease activity in association with up-regulation of the tumor necrosis factor alpha/tumor necrosis factor receptor system in systemic lupus erythematosus. *Arthritis Rheum.* (2003) 48:2533–40. 10.1002/art.11264 13130473

[74] Ronda N Favari E Borghi MO Ingegnoli F Gerosa M Chighizola C Impaired serum cholesterol efflux capacity in rheumatoid arthritis and systemic lupus erythematosus. *Ann Rheum Dis.* (2014) 73:609–15. 10.1136/annrheumdis-2012-202914 23562986

[75] Varbo A Benn M Tybjaerg-Hansen A Nordestgaard BG. Elevated remnant cholesterol causes both low-grade inflammation and ischemic heart disease, whereas elevated low-density lipoprotein cholesterol causes ischemic heart disease without inflammation. *Circulation.* (2013) 128:1298–309. 23926208 10.1161/CIRCULATIONAHA.113.003008

[76] Nordestgaard BG. Triglyceride-rich lipoproteins and atherosclerotic cardiovascular disease new insights from epidemiology, genetics, and biology. *Circ Res.* (2016) 118:547–63. 10.1161/CIRCRESAHA.115.306249 26892957

[77] Gibson T Myers AR. Subclinical liver-disease in systemic lupus-erythematosus. *Scand J Rheumatol.* (1975) 4:112. 7310775

[78] Matsumoto T Kobayashi S Shimizu H Nakajima M Watanabe S Kitami N The liver in collagen diseases: pathologic study of 160 cases with particular reference to hepatic arteritis, primary biliary cirrhosis, autoimmune hepatitis and nodular regenerative hyperplasia of the liver. *Liver.* (2000) 20:366–73. 10.1034/j.1600-0676.2000.020005366.x 11092254

[79] Runyon BA Labrecque DR Anuras S. The spectrum of liver-disease in systemic lupus-erythematosus - report of 33 histologically-proved cases and review of the literature. *Am J Med.* (1980) 69:187–94. 10.1016/0002-9343(80)90378-2 7405944

[80] Robinson G Pineda-Torra I Ciurtin C Jury EC. Lipid metabolism in autoimmune rheumatic disease: implications for modern and conventional therapies. *J Clin Invest.* (2022) 132:e148552. 10.1172/JCI148552 35040437 PMC8759788

[81] Mao Z Zhang WZ. Role of mTOR in glucose and lipid metabolism. *Int J Mol Sci.* (2018) 19:2043. 10.3390/ijms19072043 30011848 PMC6073766

[82] Laplante M Sabatini DM. mTOR signaling in growth control and disease. *Cell.* (2012) 149:274–93. 10.1016/j.cell.2012.03.017 22500797 PMC3331679

[83] Cai H Dong LLQ Liu F. Recent advances in adipose mTOR signaling and function: therapeutic prospects. *Trends Pharmacol Sci.* (2016) 37:303–17. 10.1016/j.tips.2015.11.011 26700098 PMC4811695

[84] Peterson TR Sengupta SS Harris TE Carmack AE Kang SA Balderas E mTOR complex 1 regulates lipin 1 localization to control the SREBP pathway. *Cell.* (2011) 146:408–20. 10.1016/j.cell.2011.06.034 21816276 PMC3336367

[85] Mueller MA Beutner F Teupser D Ceglarek U Thiery J. Prevention of atherosclerosis by the mTOR inhibitor everolimus in LDLR-/- mice despite severe hypercholesterolemia. *Atherosclerosis.* (2008) 198:39–48. 10.1016/j.atherosclerosis.2007.09.019 17980369

[86] Ai D Chen C Han S Ganda A Murphy AJ Haeusler R Regulation of hepatic LDL receptors by mTORC1 and PCSK9 in mice. *J Clin Invest.* (2012) 122:1262–70. 10.1172/JCI61919 22426206 PMC3314476

[87] Caron A Richard D Laplante M. The roles of mTOR complexes in lipid metabolism. *Annu Rev Nutr.* (2015) 35:321–48. 10.1146/annurev-nutr-071714-034355 26185979

[88] Kurdi A Martinet W De Meyer GRY. mTOR inhibition and cardiovascular diseases: dyslipidemia and atherosclerosis. *Transplantation.* (2018) 102(2S Suppl. 1):S44–6. 10.1097/TP.0000000000001693 28230638

[89] Murphy E. Estrogen signaling and cardiovascular disease. *Circ Res.* (2011) 109:687–96. 21885836 10.1161/CIRCRESAHA.110.236687 PMC3398381

[90] Cheskis BJ Greger JG Nagpal S Freedman LP. Signaling by estrogens. *J Cell Physiol.* (2007) 213:610–7. 17886255 10.1002/jcp.21253

[91] Ramos FJ Chen SC Garelick MG Dai DF Liao CY Schreiber KH Rapamycin reverses elevated mTORC1 signaling in lamin A/C-deficient mice, rescues cardiac and skeletal muscle function, and extends survival. *Sci Transl Med.* (2012) 4:144ra103. 10.1126/scitranslmed.3003802 22837538 PMC3613228

[92] Baar EL Carbajal KA Ong IM Lamming DW. Sex- and tissue-specific changes in mTOR signaling with age in C57BL/6J mice. *Aging Cell.* (2016) 15:155–66. 10.1111/acel.12425 26695882 PMC4717274

[93] Gurgen D Kusch A Klewitz R Hoff U Catar R Hegner B Sex-specific mTOR signaling determines sexual dimorphism in myocardial adaptation in normotensive DOCA-salt model. *Hypertension.* (2013) 61:730–6. 10.1161/HYPERTENSIONAHA.111.00276 23339165

[94] Chen J Yu J Yuan R Li N Li C Zhang X. mTOR inhibitor improves testosterone-induced myocardial hypertrophy in hypertensive rats. *J Endocrinol.* (2022) 252:179–93. 10.1530/JOE-21-0284 34874016 PMC8859925

[95] Perl A. Activation of mTOR (mechanistic target of rapamycin) in rheumatic diseases. *Nat Rev Rheumatol.* (2016) 12:169–82. 10.1038/nrrheum.2015.172 26698023 PMC5314913

[96] Lai ZW Kelly R Winans T Marchena I Shadakshari A Yu J Sirolimus in patients with clinically active systemic lupus erythematosus resistant to, or intolerant of, conventional medications: a single-arm, open-label, phase 1/2 trial. *Lancet.* (2018) 391:1186–96. 10.1016/S0140-6736(18)30485-9 29551338 PMC5891154

[97] Piranavan P Bhamra M Perl A. Metabolic targets for treatment of autoimmune diseases. *Immunometabolism.* (2020) 2:e200012. 10.20900/immunometab20200012 32341806 PMC7184931

[98] Saxton RA Sabatini DM. mTOR signaling in growth, metabolism, and disease. *Cell.* (2017) 168:960–76. 28283069 10.1016/j.cell.2017.02.004 PMC5394987

[99] Garcia M Mulvagh SL Merz CNB Buring JE Manson JE. Cardiovascular disease in women: clinical perspectives. *Circ Res.* (2016) 118:1273–93. 10.1161/CIRCRESAHA.116.307547 27081110 PMC4834856

[100] Mocumbi AO. Women’s cardiovascular health: shifting towards equity and justice. *Lancet.* (2021) 397:2315–7. 10.1016/S0140-6736(21)01017-5 34010612

[101] Vogel B Acevedo M Appelman Y Merz CNB Chieffo A Figtree GA The lancet women and cardiovascular disease commission: reducing the global burden by 2030. *Lancet.* (2021) 397:2385–438. 10.1016/S0140-6736(21)00684-X 34010613

[102] Falasinnu T Chaichian Y Bass MB Simard JF. The representation of gender and race/ethnic groups in randomized clinical trials of individuals with systemic lupus erythematosus. *Curr Rheumatol Rep.* (2018) 20:20. 10.1007/s11926-018-0728-2 29550947 PMC5857270

[103] Sepulveda JIR Bolin K Mofors J Leonard D Svenungsson E Jonsen A Sex differences in clinical presentation of systemic lupus erythematosus. *Biol Sex Differ.* (2019) 10:60. 10.1186/s13293-019-0274-2 31843005 PMC6915972

